# Laser-Induced Thermal Decomposition of Uranium Single-Source Precursors to Form Uranium Ceramics

**DOI:** 10.1021/acs.inorgchem.6c01359

**Published:** 2026-08-31

**Authors:** Sheridon N. Kelly, Andrew J. Swift, Maryline G. Ferrier, Aiden A. Martin, Jason R. Jeffries, John Arnold, Bradley C. Childs

**Affiliations:** † Physical and Life Sciences Directorate, 4578 Lawrence Livermore National Laboratory, Livermore, California 94550, United States; ‡ Department of Chemistry, 1438 University of California, Berkeley, Berkeley, California 94720, United States

## Abstract

Three molecular precursors (uranium amidinate, thioamidate, and amidate) were subjected to laser-based heating to assess how laser irradiation (*T* > 2000 °C and rapid cooling) changes uranium material formation compared to more mild conditions in furnace heating. Simultaneous thermal analysis was conducted to study mass loss and decomposition profile of the precursors under mild conditions, and suggests formation of uranium nitride, sulfide, and oxide materials. Under laser irradiation, residual gas analysis indicates more fragmentation of ligands with increasing laser power, with small gases such as N_2_, CO, and small hydrocarbons being the primary gaseous byproducts. Powder X-ray diffraction (PXRD) of the solid-state product formed from an amidinate precursor indicates formation of uranium carbide at lower laser powers, but uranium nitride at higher laser powers. PXRD for amidate and thioamidate precursors reveals nitride and carbides as major products. This suggests that the reactive environment created by the laser and gaseous byproducts is more influential than the pre-existing U–N, U–S, and U–O bonds, which are favored in furnace experiments. Overall, this study demonstrates the varied uses of these molecular precursors when subjected to differing thermal conditions, and their utility for the formation of uranium nitride and uranium carbide under laser irradiation.

## Graphical abstract

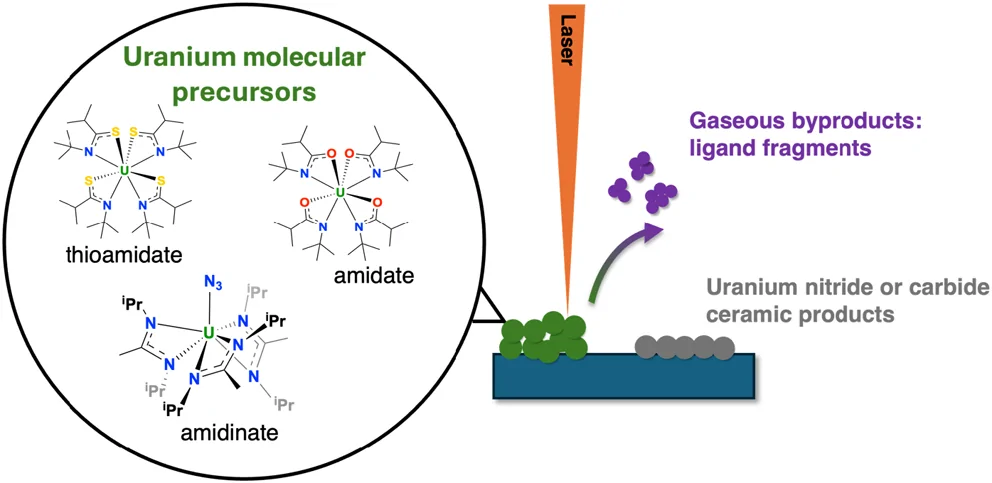

## Introduction

There has been interest in uranium-based materials since the Manhattan Project in the 1940’s. From applications in nuclear fuels^1–8^ and nuclear forensics,^9–12^ to fundamental studies of electronic structure,^13,14^ the field of uranium chemistry continues to grow. Traditionally, uranium metal has been prepared by reducing oxides or halides using electropositive elements, such as Li, Na, Mg, Ca, or Ba.^15^ Uranium oxides can be found in nature or are synthesized by oxidizing uranium metal, reducing higher oxides or halides, decomposing uranyl uranates, and other processes.^5,15^ Other uranium ceramics, such as uranium carbide and uranium nitride, are prepared typically from uranium dioxide or metal,^16,17^ and from uranium tetrafluoride.^18^ Uranium sulfides are prepared mainly by sulfurization of the oxides or metallic uranium.^19–22^ Many of these processes happen at high temperatures over extended periods of time, often with unappealing reagents, or large amounts of pyrophoric material (i.e., uranium metal). In addition to undesirable synthetic conditions, these methods lead to inconsistent chemical homogeneity and physical defects that are problematic on both a commercial scale when considering fuel lifetimes and stability^23,24^ and at a fundamental level with spectroscopic characterization.

Recently, a bottom-up approach, wherein coordination compounds are used as molecular precursors to form desirable materials, has been used to target uranium-based materials.^25–33^ This approach leads to the formation of desired materials with fewer defects, a better-controlled reaction environment, and with improved chemical homogeneity, although carbon-containing ligands often result in some carbon contamination in products.^32,34^ Previous studies from this working group have focused on developing single-source precursors to target uranium oxides^29^ and sulfides^30^ (Figure 1); these are precursors that have a preformed metal–heteroatom bond,^35^ and that, when thermally decomposed, will form the desired material. For example, the pre-existing uranium–oxygen bond in a uranium amidate molecule will remain intact as the molecule decomposes, leaving behind UO_2_.^29^ Decomposition of single-source precursors occurs at much milder conditions than are seen in typical uranium oxide and sulfide synthesis (*ca*. 100 °C for the amidate and *ca*. 210 °C for the thioamidate, compared to >950 °C in a conventional synthesis), which allows for more energy-efficient material syntheses.

**Figure 1:**
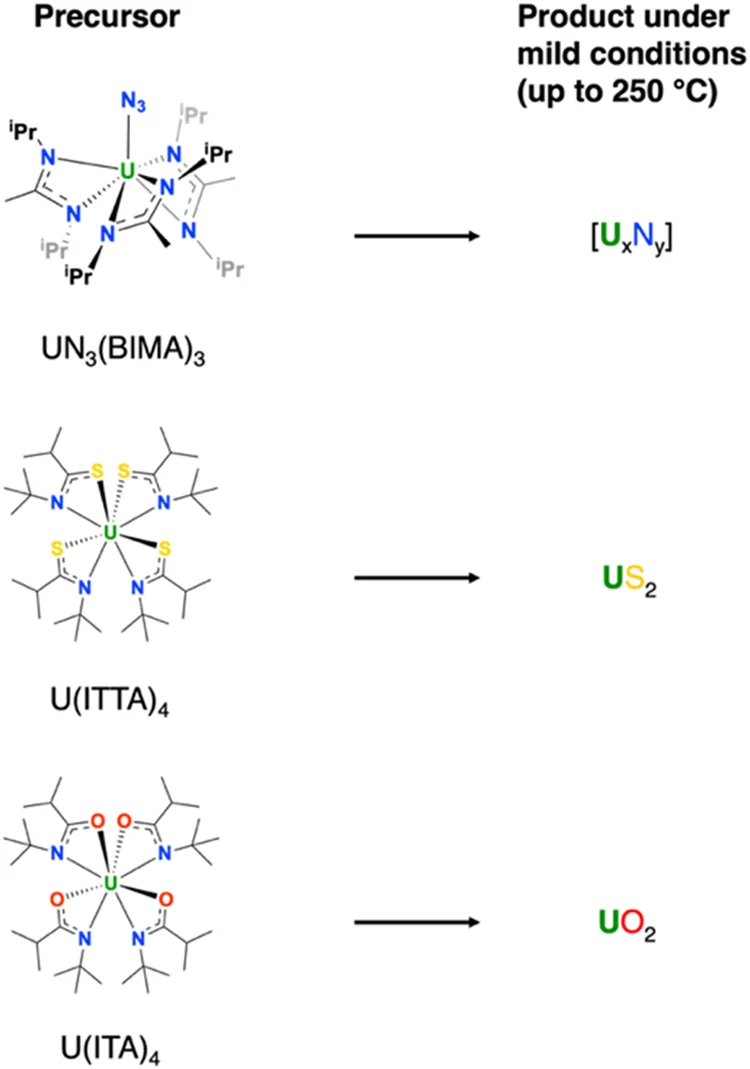
Uranium coordination complexes to be studied as precursors for uranium-based materials when subject to mild conditions (*ca.* 100–250 °C) in a tube furnace. Top shows an amidinate, UN_3_(BIMA)_3_, which has been proposed to form uranium nitride materials, although it has not been studied for this purpose in previous reports. Middle shows thioamidate U­(ITTA)_4_, which has been used as a precursor for US_2_. Bottom shows amidate U­(ITA)_4_, which has been used in a chemical vapor deposition (CVD) process to form UO_2_ thin films.

While these single-source precursors have proven effective for oxide and sulfide synthesis, uranium metal, carbide, and nitride materials have not been targeted using this approach, though they are more desirable as alternative nuclear fuel options.^36,37^ Previous work in this group has used laser irradiation with coordination complexes, namely UI_4_(1,4-dioxane)_2_,^25^ UI_3_(pyridine)_4_,^26^ UCl_4_(TMEDA)_2_,^26^ UI_3_,^28^ and (NH_4_)­UF_8_ ^28^ to form uranium metal and desirable ceramics. In these syntheses, precursors are subjected to rapid heating (up to 2600 °C in less than 1 ms) and fast cooling rates (10^6^ K/s) when irradiated with a laser.^25,26^ Overall, these studies revealed that reaction environment (Ar vs N_2_ vs CH_4_) and identity of the ligands plays a key role in determining final product. For example, carbon-rich environments favor formation of carbides, while nitrogen-rich environments favor nitride formation, even with the same starting material.^26^ Additionally, the ligand environment dictates the identity of reactive species that the uranium metal is exposed to during irradiation in the form of gaseous byproducts in the laser chamber, and thus affects the composition of the final material.^25,26^ For example, irradiation of the chloride-containing UCl_4_(TMEDA)_2_ produced less uranium carbide than the iodide-containing UI_3_(pyridine)_4_ because the chlorine reacted more readily with the carbon-containing byproducts.^26^ Simple halogen-based ligands are advantageous for these kinds of syntheses given that the halogens act as excellent leaving groups, but the reactivity of their byproduct gases limits synthetic scope.

Since previous literature with amidates and thioamidates have demonstrated that nonhalogen-based ligands can also serve as excellent “leaving groups” while simultaneously conferring synthetic control over the product, we sought to study these single-source precursors by laser irradiation. In this work, we report the thermal decomposition of three uranium coordination complexes with N, O, and S coordination shells via laser-based heating. All precursors were studied under an inert argon atmosphere at various laser conditions in order to determine the influence of the ligands in creating a reactive environment when subject to laser irradiation. Simultaneous thermal analysis (STA) was conducted to compare differences in thermal behavior when subjected to mild vs laser environments. A residual gas analyzer (RGA) was connected to the laser chamber to study fragmentation of the ligands, and postirradiation products were studied with powder X-ray diffraction (PXRD).

## Results and Discussion

The three precursors were synthesized according to literature methods.^29,30,38^ Following isolation, the complexes were subjected to STA, which offers initial insight into the chemistry of the precursors and how they might decompose to form nitrides, sulfides, and oxides (Figure 2).

**Figure 2:**
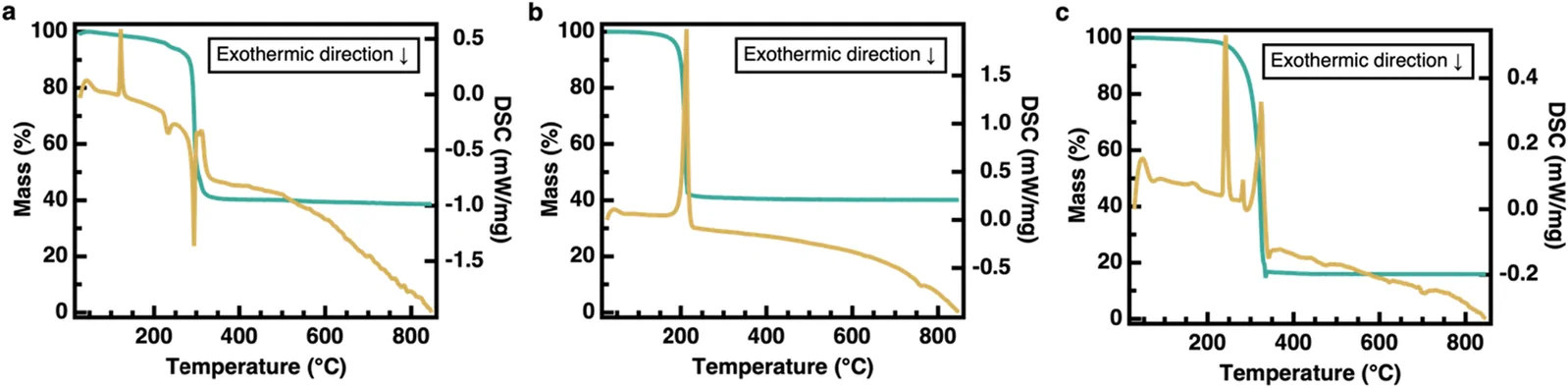
Simultaneous thermal analysis of all precursors collected at a heating rate of 5 °C/min up to 850 °C. The thermogravimetric analysis (TGA) is shown in green and differential scanning calorimetry (DSC) is shown in tan. Image (a) shows UN_3_(BIMA)_3_, with a mass loss of 60.7%. DSC shows 3 steps: an endothermic peak at 122 °C, an exothermic peak at 235 °C, and an exothermic peak at 296 °C. Image (b) shows U­(ITTA)_4_, with a mass loss of 58.6%. DSC includes a single endothermic peak at 213 °C. Image (c) shows U­(ITA)_4_, with a mass loss of 83%. DSC shows three endothermic peaks, one at 242 °C, one at 282 °C, and one at 324 °C.

### Simultaneous Thermal Analysis (STA)

Thermogravimetric analysis (TGA) for UN_3_(BIMA)_3_ reveals a mass loss of 60.7% (Figure 2a), which is consistent with loss of three BIMA ligands (calcd: 60.5%), suggesting formation of a uranium nitride species when the uranium metal center and the nitrogen atoms of the N_3_^–^ ligand remain. Thermal decomposition experiments of group 11 and 13 amidinate species have revealed two likely pathways for decomposition: carbodiimide deinsertion, wherein a M–C bond remains, and β-hydrogen elimination, wherein free ligand is lost and an M–H bond remains.^39,40^ In the first case, a UCN complex would be formed, with an expected mass loss of 62%. When considering the second pathway, a UN_*x*_H_*y*_ species is not reported, and the mass loss does not suggest the formation of UH_3_ (calcd: 66%), so it is more likely that a U_*x*_N_*y*_ species is formed. Studies of lanthanide amidinates as single-source precursors typically result in metal nitride materials.^41–43^ Differential scanning calorimetry (DSC) for UN_3_(BIMA)_3_ is more complicated, revealing an endothermic peak at 122 °C with 1.3% mass loss, a small exothermic peak at 235 °C with 6.8% mass loss, and a larger exothermic peak at 296 °C corresponding to the largest mass loss of 60.7%. The reported melting point (simultaneous with decomposition) for UN_3_(BIMA)_3_ is *ca*. 272 °C, suggesting that the observed peak at 295.9 °C is associated with decomposition of the molecule. The smaller peak at 122 °C may be a small amount of toluene (which was used in compound synthesis) that is removed as the sample is heated. The peak at 235 °C may be indicative of offgassing of small gaseous byproducts, such as N_2_ or nitrous oxides if any oxide is present in the system.

Thermal analysis for U­(ITTA)_4_ shows a single endothermic peak at 213 °C associated with decomposition of U­(ITTA)_4_, with a mass loss measured at 238 °C of 58.6% (Figure 2b). As previously reported, this is less than the predicted mass loss of 65.3%, suggesting the incorporation of carbon impurities in the final product. The decomposition of U­(ITTA)_4_ is associated with loss of isobutylene, isobutyronitrile, and the protonated H­(ITTA) ligand, leaving sulfide bound to the uranium metal center to form US_2_.^30^ Meanwhile, STA for U­(ITA)_4_ reveals an overall mass loss of 83%, which is higher than the predicted 66% for the decomposition of U­(ITA)_4_ into UO_2_ (Figure 2c). U­(ITA)_4_ is volatile, however, so additional mass loss may be attributed to sublimation of the precursor prior to decomposition.^29^ DSC for U­(ITA)_4_ shows multiple endothermic steps: one at 242 °C with a mass loss of 3%, a smaller peak at 282 °C with 10% mass loss, and another large peak at 324 °C associated with the largest mass loss of 83%. The presence of multiple peaks suggests that decomposition of the precursor occurs in multiple steps, as reported by Straub et al.^29^

Overall, behavior of the precursors at mild heating conditions demonstrates the utility of these single-source precursors for forming desirable uranium-containing materials at relatively low temperatures. All precursors decompose below *ca*. 350 °C, with no further mass loss up to 850 °C, indicating full conversion at lower temperatures.

### Residual Gas Analysis (RGA)

When shifting to laser irradiation studies, we expect to see much higher temperature and pressure gradients, leading to the simultaneous decomposition of the whole sample and formation of reactive gaseous fragments. To understand how these conditions affect decomposition compared with mild conditions, RGA was used to assess gaseous products during laser irradiation. A residual gas analyzer was connected to the laser chamber, and experiments were conducted under high-vacuum conditions in order to assess what gases were produced by the precursors themselves. All precursors were subject to the laser conditions described in the Experimental Section, and RGA data was collected for a minute before irradiation and up to 3 min after in order to understand the baseline for the gases.

As each precursor contains a substantial amount of nitrogen and carbon, a mass of 28 amu, which is associated with N_2_, CO, and C_2_H_4_, was analyzed. The peak at 28 amu was the most prevalent of all masses measured for each precursor at each laser condition, with partial pressure change on the order of 10^–6^ Torr (Figure 3 for 50 W measurements, Figures S2, S4, S6 for all other laser conditions), indicating that N_2_, CO, and/or C_2_H_4_ were the largest gaseous products for each precursor at low and high laser power settings; this suggests that conditions created by laser irradiation lead to more fragmentation and thus smaller fragments for each precursor compared to that observed at mild conditions in a furnace. The peak at 28 amu may be most prevalent because there are multiple species contributing to the change in partial pressure, rather than contributions from a single species.

**Figure 3:**
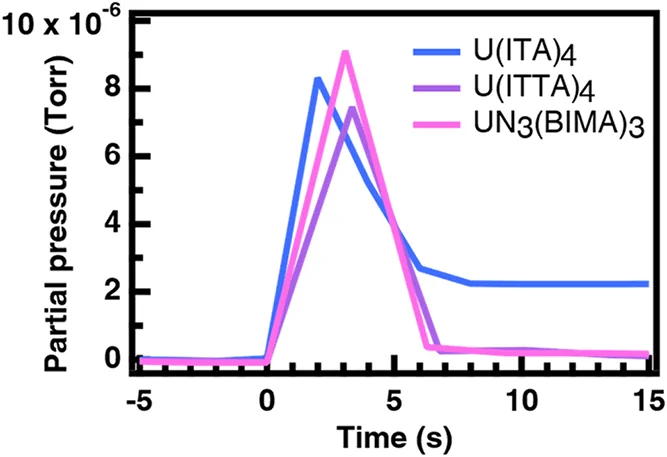
Partial pressure as a function of time for 28 amu (corresponding to N_2_, CO, or C_2_H_4_) captured with a residual gas analyzer (RGA). Time = 0 corresponds to the time when the laser was turned on. A single laser scan was performed over a 0.9 × 0.9 mm^2^ box using 50 W power.

To understand the decomposition of UN_3_(BIMA)_3_, we looked to previous thermal degradation studies of similar amidinate ligands. Studies indicate that amidinate compounds can decompose following a carbodiimide deinsertion pathway or a β-hydrogen elimination pathway, leaving behind the free ligand: in this case, the products would be *^i^* Pr–N=C=N–*^i^* Pr (128 amu) and BIMA (143 amu).^39^ Other smaller fragments have been observed in electron-impact mass spectrometry studies of amidinate compounds, including (iso)­nitriles (such as *^i^* PrNC^+^) and hydrocarbons (such as *^i^* Pr^+^).^44^ Based on these studies, the masses represented in the Supporting Information (Figure S3) were measured with RGA (Figure 4a). With one 50 W pulse, the largest mass fragments (59, 87, and 143 amu) measured were observed only in very small amounts, suggesting that high laser energy leads to more fragmentation and that larger fragments are not stable in this environment. Fragments associated with 42 amu (N_3_ and C_3_H_6_) and 43 amu (HN_3_ or C_2_H_5_N) were the most prevalent after 28 amu. These masses are associated with the loss of the metal-bound N_3_ group (which was not anticipated following STA studies) and fragmentation of the BIMA ligand into smaller hydrocarbons. Partial pressure changes for 16, 17, 26, and 30 amu, all associated with other hydrocarbon and N-containing compounds, further suggest the fragmentation of the BIMA ligand into smaller components. Similar fragmentation and changes in partial pressure of each mass were observed when the compound was irradiated at 100 W (Figure S2).

**Figure 4:**
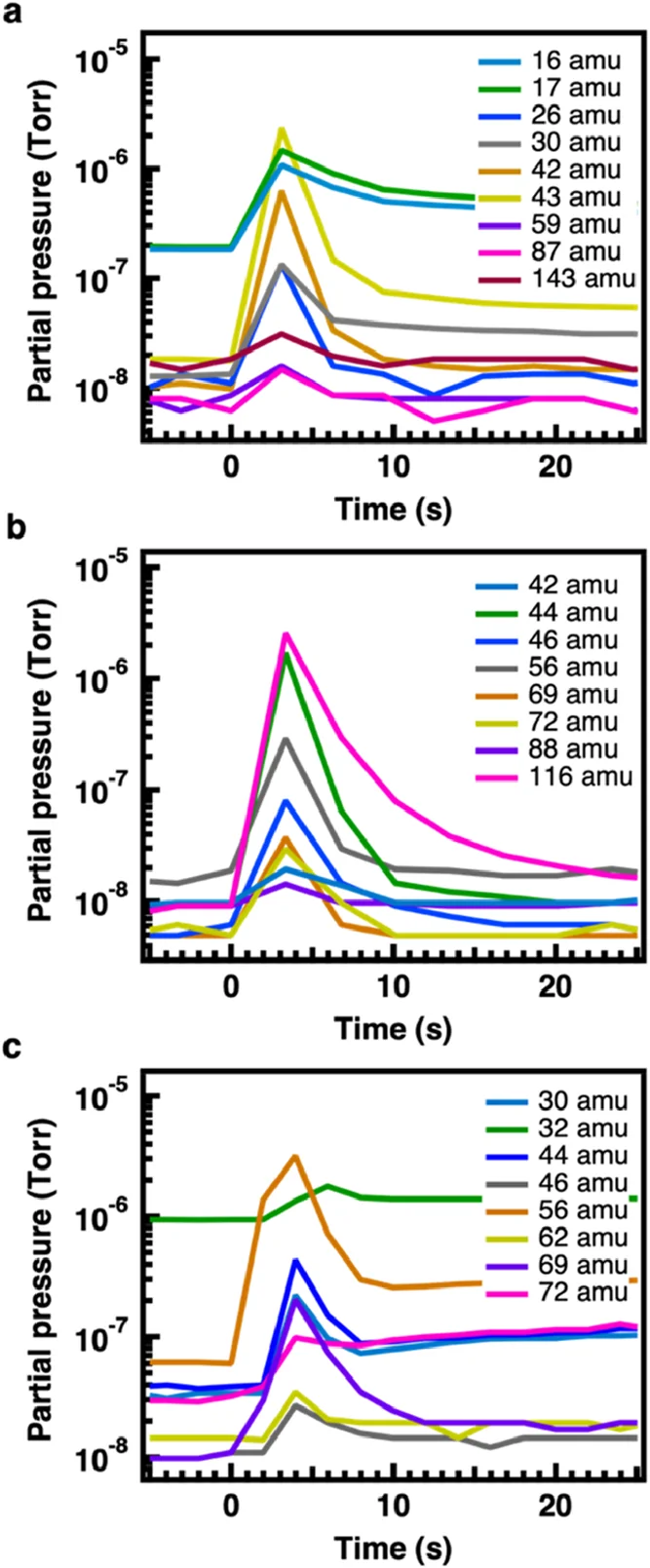
Partial pressure as a function of time for various gas species captured by RGA. Time = 0 corresponds to the time when the laser was turned on. The logarithmic scale of the *y*-axis highlights the order of magnitude for partial pressure changes of each mass. A single laser scan was performed over a 0.9 × 0.9 mm^2^ box using 50 W power. (a) Irradiation of UN_3_BIMA_3_. (b) Irradiation of U­(ITTA)_4_. (c) Irradiation of U­(ITA)_4_.

A sequence of increasing laser irradiation on the same area was performed: 10 W, followed by a 20 W pulse, followed by a second 20 W pulse (henceforth termed the 10, 20, 20 W sequence). Overall, the same fragmentation was observed for this sequence as for the 50 and 100 W experiments (Figure S2). The 42 and 43 amu partial pressures increased from 10 to 20 W to 20 W, suggesting that higher power or multiple irradiations are necessary for loss of the bound N_3_ group or further fragmentation of the BIMA ligand. On the other hand, the peak associated with 30 amu is present in the 10 W scan, less so in the first 20 W pulse, and was not observed for the second 20 W pulse, suggesting that this slightly larger hydrocarbon (C_3_H_6_) is one of the first fragments formed but is not formed again or is further fragmented with subsequent laser pulses.

In studies for U­(ITTA)_4_ and U­(ITA)_4_, we turned to our previous work on the thermal decomposition of these precursors in sealed NMR tubes to understand potential fragmentation.^29,30^ These experiments indicate the formation of isobutylene (56 amu), isobutyronitrile (69 amu), and free amide or thioamide (143 and 159 amu, respectively). Thus, we studied these masses with RGA, as well as masses associated with other possible fragments, namely, hydrocarbons, and other N-, O-, or S-containing compounds (Figures 4 and S4–S7). For the 50 W experiment, the free amidate (H­(ITA)) and thioamidate (H­(ITTA)) were not observed, implying more fragmentation and thus smaller fragments than observed in low-temperature experiments.

Looking specifically at U­(ITTA)_4_ (Figure 4b), the most prevalent peak after 28 amu is at 116 amu, which is proposed to be associated with a fragment of the free ligand without the isopropyl group (Figure S5). This is the largest fragment that is prevalent for any of the compounds, suggesting that the thioamide does not break down as much as the other precursors. Partial pressure changes for the 44, 46, and 56 amu channels were the other most prevalent peaks for U­(ITTA)_4_ irradiated at 50 W. The peak at 44 amu is likely associated with CS or C_3_H_8_; 46 amu with NS, CH_2_S, or CH_6_N_2_; and 56 amu with C_4_H_8_, which are all fragments of the H­(ITTA) ligand or likely products in the reactive environment created by the rapid heating of irradiation (in the case of NS). Larger masses such as 69, 72, and 88 amu were observed only in minor amounts, suggesting that either the largest fragment (116 amu) does not further break apart or that if it does, smaller masses are preferred. The 100 W pulse experiment is consistent with the 50 W experiment, suggesting that an increased laser power does not further affect the fragmentation of U­(ITTA)_4_ (Figure S4).

When subjecting U­(ITTA)_4_ to a 10, 20, and 20 W sequence, fewer masses changed in partial pressure over the course of data collection (Figure S4). With one 10 W pulse, 42 and 56 amu were observed as the most prevalent masses, even more than 28 amu. These are both associated with hydrocarbons (C_3_H_6_ and C_4_H_8_), suggesting that carbon substituents on the ligand undergo fragmentation but that perhaps the U–N and U–S bonds remain intact. With the first 20 W pulse, changes in partial pressure for 44 and 116 amu were observed, associated with the CS or C_3_H_8_ and the larger ligand fragment observed in the 50 W experiment. This suggests that slightly higher power than 10 W is necessary for removing the larger S-containing fragments yet is not enough to lead to further, mixed fragmentation. The second 20 W pulse yielded no change in the partial pressures for any masses, suggesting that all fragmentation and reactions occurred with the first two pulses.

For the amidate, U­(ITA)_4_, at a 50 W laser pulse, middling masses of 44, 56, and 69 amu are observed as the most prevalent peaks (Figures 4c and S6, S7). These are associated with hydrocarbons, CO_2_, N_2_O, as well as isobutylene and isobutyronitrile (which are both observed in the low-temperature decomposition experiments reported by Straub et al.^29^). These all point to some amount of ligand fragmentation further when subject to the high power of the laser as compared to mild heating in a furnace. There is some pressure change measured for the 30 amu measurement and minimal change for 32, 46, 62, and 72 amu peaks, indicating that very little of the compounds associated with those masses are formed. The 100 W experiment reflects the same observations as those of the 50 W experiment (Figure S6).

For the 10, 20, and 20 W sequences, changes in partial pressures for all masses are similar to that observed in the 50 and 100 W experiments, with the exception of 143 amu (Figure S6). There is a slight change in partial pressure for 143 amu, associated with the protonated amide, H­(ITA), which was not observed for the higher-power experiments. This indicates that lower power does not lead to fragmentation of all of the ligand into smaller parts as the higher powers must.

Overall, RGA experiments indicate that a large amount of N_2_, CO, or C_2_H_4_ is produced for all of the samples. Additionally, ligands around each sample fragment primarily into much smaller pieces than those observed in previous thermal decomposition experiments. The difference in gaseous byproducts suggests changes in the chemistry of the reaction environment as compared to more mild conditions in a tube furnace and indicates that the final solid-state product will be different as the precursors decompose differently.

### Powder Analysis

After studying precursor fragmentation with RGA, precursors were subjected to various laser conditions to study the irradiated products. Figure 5 shows images for the precursors before irradiation and postirradiation products for the 10, 20, 20 W sequence, and for 50 and 100 W laser experiments. All post-irradiation samples have a dark-colored layer of solid adhered to the substrate. Some samples, namely, for the 50 W experiments, had some beaded sample on the surface. For most samples, there is some precursor outside of the laser zone visible to varying degrees of decomposition (i.e., some green precursor visible).

**Figure 5:**
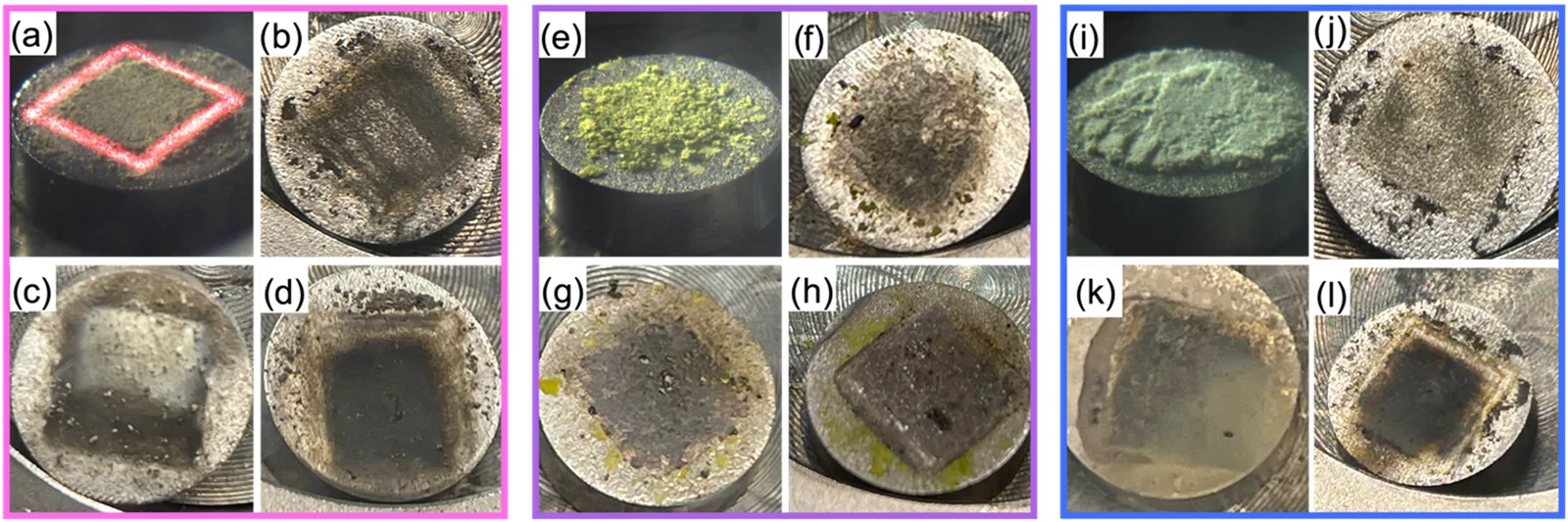
Optical images of samples before and after irradiation. All precursors are a shade of green before irradiation, and images were captured by a camera pointed into the laser chamber. After irradiation, images were taken inside a glovebox. Some samples show beads on the surface while others show a thin gray layer of solid adhered to the substrate. Images (a)–(d), boxed in pink, are for UN_3_BIMA_3_. Images (e)–(h), boxed in purple, are for U­(ITTA)_4_. Images (i)–(l), boxed in blue, are for U­(ITA)_4_.

UN_3_BIMA_3_ was irradiated with two 10 W pulses, and the powder pattern for this sample aligns with that of UC with no additional peaks (Figure 6a).^45^ Formation of uranium carbonitride phases cannot be excluded, as typical powder diffraction techniques do not distinguish between these ceramic phases. Although mild thermal decomposition conditions in STA experiments suggested loss of BIMA ligands and formation of a U–N complex, this was not observed under laser irradiation. Formation of carbide species results from the environment created by laser irradiation leading to reactivity with gaseous hydrocarbon species that are produced by ligand fragmentation, thus forming uranium carbide despite the precursor’s many U–N bonds. Similar results were seen previously, where the presence of outer-sphere organic ligands was more influential to the final product than coordinated heteroatoms, leading to the formation of uranium carbide and oxide species.^25,46,47^

**Figure 6:**
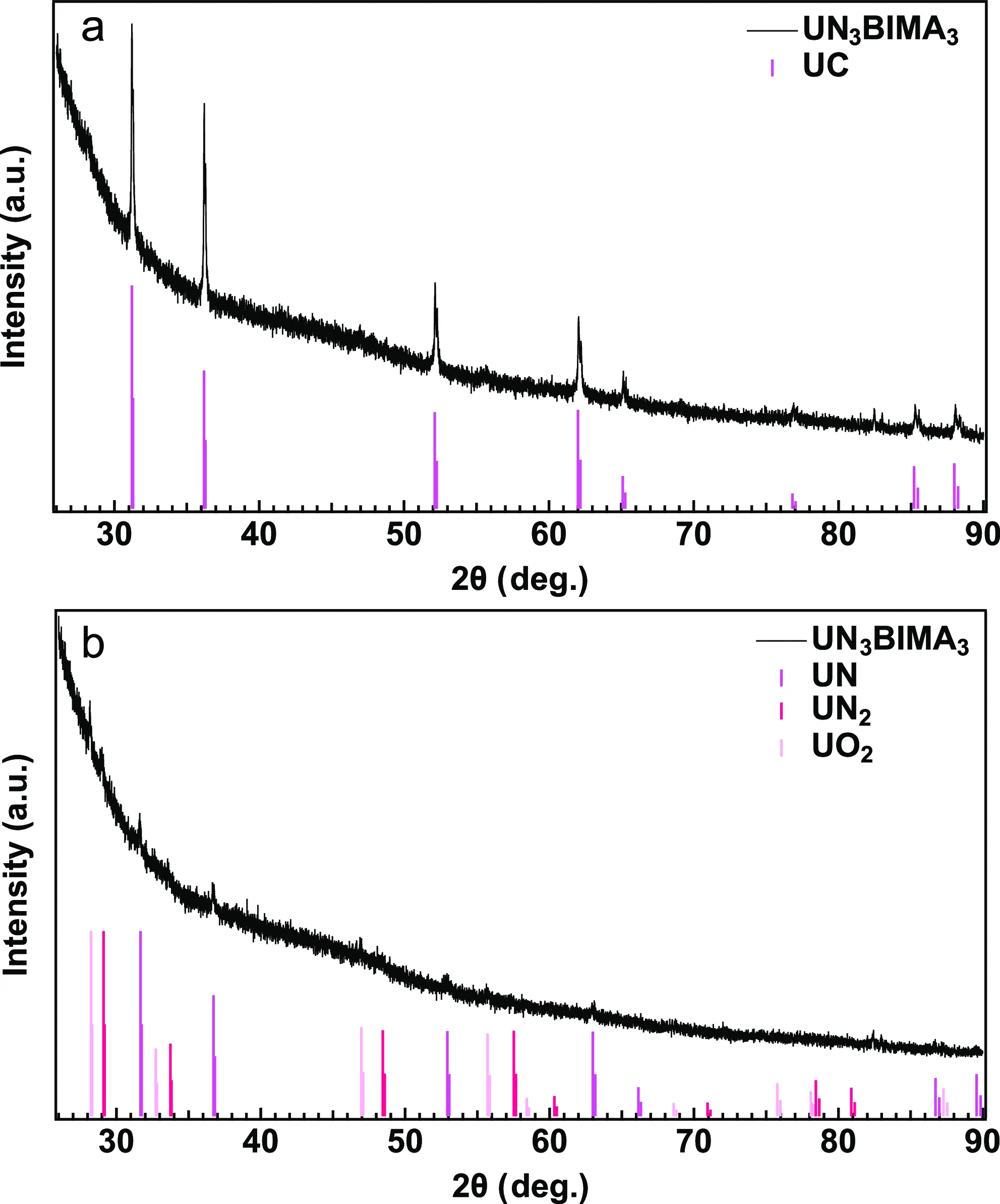
Powder X-ray diffraction for UN_3_BIMA_3_ irradiated with (a) two 10 W pulses and (b) two 10 W pulses, a 20 W pulse, and two 50 W pulses in sequence. Both powder patterns are presented in black and are compared to their major products in pink.

When subjecting UN_3_BIMA_3_ to the same two 10 W pulses, followed by higher laser powers (a sequence of 10, 10, 20, 50, 50 W pulses), uranium nitride species (UN and UN_2_) are observed (Figure 6b, Table S1), while UC is not identified in the powder pattern following Rietveld refinement. This implies that, while UC is formed with the first two low-powered pulses, further reactivity with a nitrogen-rich atmosphere (see: RGA experiments above) converts the originally produced UC to nitride when more energy is provided to the system, an observation supported by previous work on carbothermic reduction of UC to UN.^48–51^ Alternatively, this could mean that there were amorphous nitride species that were unobservable in the 10, 10 W sequence that was crystallized with higher laser power. The ability to exactly quantify carbon and nitrogen content is limited by the lack of sensitivity of energy dispersive X-ray spectroscopy (EDS) to distinguish carbon and nitrogen, and only small amounts of product (as seen in Figure 5) prevented characterization through other methods.

Similar conditions were applied to U­(ITTA)_4_ and U­(ITA)_4_. A 10, 10 W laser sequence did not lead to diffraction for either precursor. The second sequence (10, 10, 20, 50, 50 W) that was applied to the amidinate did lead to some diffraction for both products (Figure S8, Table S2). Low-intensity peaks in these powder patterns are likely a result of low crystallinity or small sample size, owing to the intentional design of these precursors to sublimate and react at low temperatures (<300 °C).^29,30^ In the case of U­(ITTA)_4_ (Figure S8a), the most intense peaks align with UC, followed by UN, U_2_N_3_, and UO_2_. Carbide and nitride species are a result of the gaseous nitrogen and carbon-containing products as seen in RGA experiments. Presence of UO_2_ in this sample would be indicative of trace O_2_ or oxygen-containing products. The most intense peaks in the pattern generated from laser irradiation of U­(ITA)_4_ (Figure S8b) are UN, UO_2_, and U_2_N_3_. This may be a result of the existing U–N and U–O bonds in the precursor, or a result of a reactive, nitrogen- and oxygen-rich atmosphere produced by the decomposition of the precursor. Uranium carbide was not identified in this pattern, indicating none of the carbon-containing byproducts reacted with the precursor, or that, as suggested with UN_3_BIMA_3_, UC reacted to form uranium nitrides.

## Conclusions

This work reports the study of three uranium molecules with different coordination spheres as precursors for uranium materials. Under mild thermal conditions, such as those used in furnace heating (100–850 °C), STA experiments imply the formation of uranium nitride, sulfide, and oxide materials based on the loss of mass correlating to the outer sphere of the ligands. When subjected to higher temperatures and gaseous fragments with laser heating, the results are different. RGA experiments demonstrated that the higher power of the laser leads to more fragmentation of the molecules: smaller hydrocarbons and gases such as N_2_ and CO were the most prevalent gaseous products upon decomposition. Previous studies of U­(ITA)_4_ and U­(ITTA)_4_ identified larger fragments as byproducts, indicating that the conditions experienced under laser irradiation changes the decomposition behavior and gaseous environment as compared to mild furnace conditions.^29,30^ Additionally, PXRD studies of the final solid-state products indicate the formation of uranium carbide and nitride, a deviation from what was observed in furnace experiments. Previous work has demonstrated the maintenance of the U–O or U–S bond in the ligands of U­(ITA)_4_ and U­(ITTA)_4_, an indicator also that the U–N bond in UN_3_BIMA_3_ would remain intact. Instead, the higher temperature and reactive gas environment seem to play a larger role under laser irradiation. The many hydrocarbons produced in the gaseous environment, arising from many carbon substituents on the ligands, react with the uranium material to produce uranium carbide, while nitrogen in ligand fragments generates nitride phases. The differing results under furnace vs laser-based heating demonstrate the versatile utility of molecular precursors to target varied materials when exposed to different thermal conditions: precursors developed to form uranium chalcogenides instead produce carbides and nitrides when subject to laser-based heating. Future work will focus on studying these precursors under different gas environments, as well as the development of molecular precursors with noncarbon ligand substituents to understand the role that noncarbon gaseous byproducts can play in material formation.

## Experimental Section

### Methods and Materials

#### General Considerations

Unless otherwise noted, all reactions were performed using standard Schlenk line techniques under an atmosphere of nitrogen, or in an inert atmosphere glovebox under an atmosphere of nitrogen or argon. Glassware, Celite, and cannulae were stored in an oven at ca. 160 °C for at least 4 h prior to use. Three and 4 Å molecular sieves were activated by heating under vacuum at 300 °C for 24 h.

**Caution!** U-238 is a weak α-emitter, which poses a hazard to human health; manipulations and reactions were carried out in monitored fume hoods or in inert atmosphere glove boxes in a laboratory equipped with α- and β-counting equipment.

#### Materials

*n*-Hexane, toluene, and tetrahydrofuran were purified by passage through a column of activated alumina prior to use. U­(ITA)_4_,^29^ U­(ITTA)_4_,^30^ and UN_3_(BIMA)_3_ ^38^ were synthesized according to the literature procedures. All other chemicals were purchased from commercial sources and used as received.

#### Simultaneous Thermal Analysis (STA)

Simultaneous thermal analysis (STA), which performs thermogravimetric analysis (TGA) and differential scanning calorimetry (DSC) in tandem, measurements were performed on UN_3_(BIMA)_3_ and U­(ITA)_4_ to study material thermal decomposition. Samples of ∼5–20 mg were transferred and loaded in a positive-pressure argon glovebox, which is dedicated only for STA measurements. Prior to sample analysis, the instrument was temperature and weight calibrated with a series of metal calibrants. STA measurements were performed on a NETZSCH STA 449F1-Jupiter. The sample chamber was purged and flushed with argon three times to ensure an inert atmosphere prior to sample heating. Samples were heated to 850 °C in Al_2_O_3_ crucibles at a constant heating rate of 5 °C/min. TG mass loss and DSC heat output curves were generated using NETZSCH 6.1.0 Proteus software.

Approximately 45 mg of green precursor was loaded into an Al_2_O_3_ crucible in an inert atmosphere glovebox. The sample was then transferred to a tube furnace and heated under Ar at 250 °C for 1 h, then annealed at 850 °C for 5 h. After cooling to room temperature, the sample was retrieved in an inert atmosphere transfer container and returned to a glovebox.

#### Laser Irradiation

Titanium alloy substrates (Ti-6Al-4 V, Ti with 5 wt % Al and 4 wt % V) were used for laser irradiation experiments based on previous work conducted by the group, wherein uranium-containing molecules did not react with the substrate.^25,26^ Substrates were cleaned via laser irradiation to remove a surface oxide layer. Cleaned plates were then brought into the glovebox, where samples were prepared under an argon atmosphere. Irradiation of molecular precursors was performed under an argon (ultrahigh-purity, Airgas, Inc.) environment. The laser irradiation setup and experimental conditions are described in detail elsewhere.^25^ To summarize, the laser chamber was set to 675 Torr prior to each experiment, and argon flowed over the sample to maintain an inert atmosphere. Irradiations were performed with a 1070 nm, continuous-wave fiber laser and a galvanometer-based scanning mirror system. The beam diameter was approximately 530 μm, scan speeds were set to 500 mm/s, and line scans with 75 μm spacing between lines were used. Each sample was pressed into a ∼5 × 5 mm^2^ onto the Ti64 substrate. For the settings with multiple scans, the chamber was evacuated after each scan with time for gaseous byproducts to clear before performing subsequent scans. Simulations of thermal profiles conducted in previous work on the same substrate and laser parameters indicate that a Ti64 substrate heats to 800 °C, while sample surfaces rapidly heat to >2000 °C during irradiation, overall indicating that precursors are subject to extreme thermal gradients.^25^ Once irradiation concluded, the chamber was put under partial vacuum, sealed, and transferred to an argon-filled glovebox.

#### Residual Gas Analysis (RGA)

In situ residual gas analysis was performed for each compound under high-vacuum conditions using a benchtop residual gas analyzer (RGA, UGA300, Stanford Research Systems). The sample was placed in the laser chamber, which was then connected to the vacuum system for the RGA. The chamber was evacuated overnight to ∼1.5 × 10^–6^ Torr with a turbo molecular pump. Partial pressures of selected masses ranging from 2 to 159 atomic mass units (amu) were recorded during laser irradiation as a function of time using a time step of ∼3 s per data point. A sample area of 0.9 × 0.9 mm^2^ was irradiated with the following laser settings:

One area was scanned once at 10 W, then 20 W, and then 20 W, with the RGA being recorded separately for each scan on the same area.

Another area had one scan at 50 W.

A third area was scanned with one scan at 100 W.

These laser settings closely mimic the conditions of laser irradiation.

#### Powder X-ray Diffraction (PXRD)

Samples were collected post-laser irradiation in an inert atmosphere on a zero-background Si plate PXRD holder and affixed with Kapton tape. The holders were then enclosed in an airtight dome. A D8 Discover X-ray powder diffraction (Bruker) instrument with a Cu anode was used to collect PXRD patterns at 40 kV and 40 mA power. The data were analyzed by Rietveld refinement using the general structure analysis system (GSAS).^52^

## Supplementary Material



## Notes

Experimental details, additional RGA and PXRD data (PDF)
