# Supplementary material for: Laser-Induced Thermal Decomposition of Uranium Single-Source Precursors to Form Uranium Ceramics
Source: Inorg Chem. 2026 Aug 31;65(36):20805–13. doi: 10.1021/acs.inorgchem.6c01359 (PMC13580132; doi:10.1021/acs.inorgchem.6c01359)
Supplement: Supplementary file 1 [file ic6c01359_si_001.pdf]

Supplementary Information

for:

Laser-induced thermal decomposition of uranium single-source precursors to form uranium ceramics

Sheridon N. Kelly,[a] Andrew J. Swift,[a] Maryline G. Ferrier,[a] Aiden A. Martin[a], Jason R. Jeffries,[a] John Arnold,[b] Bradley C. Childs[a*]

[a]*Physical and Life Sciences Directorate, Lawrence Livermore National Laboratory, Livermore, CA 94550, USA*
[b]*Department of Chemistry, University of California, Berkeley, Berkeley, CA 94720, USA*
* Email: childs4@llnl.gov

Table of Contents

A. Experimental Section……….…………………………………………...………….…………….....S2
B. Residual Gas Analysis……..………………………………………………...………………….....S3
C. Powder X-Ray Diffraction…….………………………..………...…..……..........................S7
D. References…………………….…………………………………......…..…….........................S8

A. Experimental Section

Methods and Materials

General Considerations: Unless otherwise noted, all reactions were performed using standard Schlenk line techniques under an atmosphere of nitrogen, or in an inert atmosphere glovebox under an atmosphere of nitrogen or argon. Glassware, Celite, and cannulae were stored in an oven at ca. 160 °C for at least 4 h. prior to use. 3 Å and 4 Å molecular sieves were activated by heating under vacuum at 300 °C for 24 h.

Caution! U-238 is a weak α-emitter which poses a hazard to human health; manipulations and reactions were carried out in monitored fume hoods or in inert atmosphere glove boxes in a laboratory equipped with α- and β-counting equipment.

Materials: *n*-Hexane, toluene, and tetrahydrofuran were purified by passage through a column of activated alumina prior to use. $U(ITA)_4$,[1] $U(ITTA)_4$,[2] and $UN_3(BIMA)_3$[3] were synthesized according to literature procedures. All other chemicals were purchased from commercial sources and used as received.

Simultaneous Thermal Analysis (STA):

Simultaneous thermal analysis (STA), which performs thermogravimetric analysis (TGA) and differential scanning calorimetry (DSC) in tandem, measurements were performed on $UN_3(BIMA)_3$ and $U(ITA)_4$ to study material thermal decomposition. Samples of ~5-20 mg were transferred and loaded in a positive pressure argon glovebox, which is dedicated only for STA measurements. Prior to sample analysis, the instrument was temperature and weight calibrated with a series of metal calibrants. STA measurements were performed on a NETZSCH STA 449F1-Jupiter. The sample chamber was purged and flushed with argon three times to ensure inert atmosphere prior to sample heating. Samples were heated to 850 °C in Al_2O_3 crucibles at a constant heating rate of 5 °C/min. TG mass loss and DSC heat output curves were generated using NETZSCH 6.1.0 Proteus® software.

Laser irradiation

Titanium alloy substrates (Ti-6Al-4V, Ti with 5 wt% Al and 4 wt% V) were used for laser irradiation experiments based on previous work conducted by the group, wherein uranium-containing molecules did not react with the substrate.[4,5] Substrates were cleaned *via* laser irradiation to remove a surface oxide layer. Cleaned plates were then brought into the glovebox, where samples were prepared under an argon atmosphere. Irradiation of molecular precursors was performed under argon (ultrahigh-purity, Airgas, Inc.) environment. The laser irradiation setup and experimental conditions are described in detail elsewhere.[4] To summarize, the laser chamber was set to 675 Torr prior to each experiment, and argon flowed over the sample to maintain an inert atmosphere. Irradiations were performed with a 1070 nm, continuous-wave fiber laser and a galvanometer-based scanning mirror system. The beam diameter was approximately 530 μm, scan speeds were set to 500 mm/s, and line scans with 75 μm spacing between lines were used. Each sample was pressed into a ~5 x 5 mm square onto the Ti64 baseplate. For the settings with multiple scans, the chamber was evacuated after each scan with time for gaseous byproducts to clear before performing subsequent scans. Once irradiation concluded, the chamber was put under partial vacuum, sealed, and transferred to an argon-filled glovebox.

Residual Gas Analysis (RGA):
In situ residual gas analysis was performed for each compound under high-vacuum conditions using a benchtop residual gas analyzer (RGA, UGA300, Stanford Research Systems). The sample was placed in the laser chamber, which was then connected to the vacuum system for the RGA. The chamber was evacuated overnight to ~1.5 × 10^{-6} torr with a turbo molecular pump. Partial pressures of selected masses ranging from 2 to 159 atomic mass units (amu) were recorded during laser irradiation as a function of time using a time step of ~3 s per datapoint. A sample area of 0.9 × 0.9 mm was irradiated with the following laser settings:
1 area scanned once at 10 W, then 20 W, then 20 W, with the RGA being recorded separately for each scan on the same area.
Another area with one scan at 50 W.
A third area with one scan at 100 W.
These laser settings closely mimic the conditions for laser irradiation.

Powder X-ray Diffraction (PXRD)
Samples were collected post-laser irradiation in an inert atmosphere on a zero-background Si plate PXRD holder and affixed with Kapton tape. The holders were then enclosed in an airtight dome. A D8 Discover X-ray powder diffraction (Bruker) with a Cu anode was used to collect PXRD patterns at 40 kV and 40 mA power. The data was analyzed by Rietveld refinement using the general structure analysis system (GSAS).[6]

B. Residual Gas Analysis

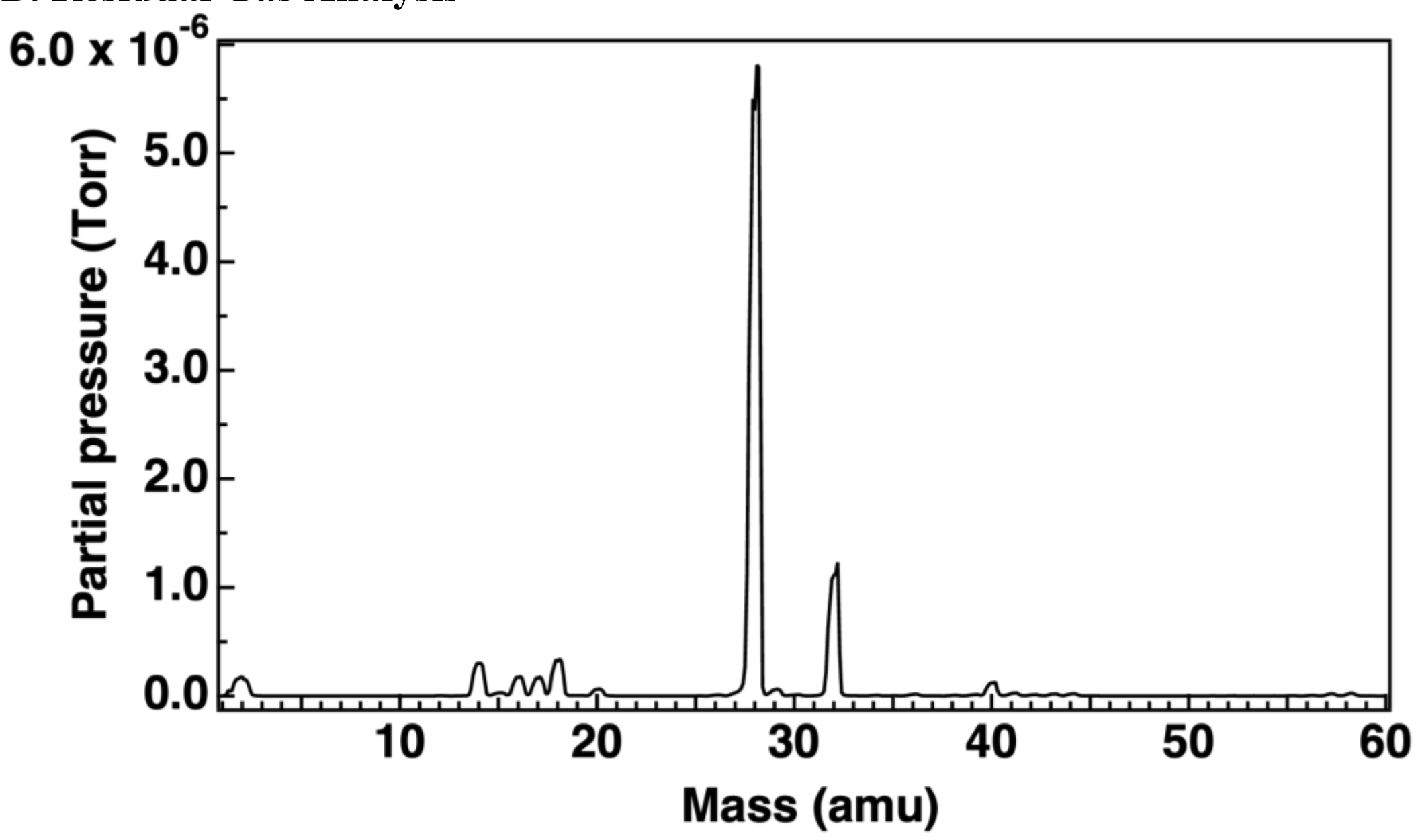

Figure S1. Partial pressure as a function of mass prior to laser irradiation of uranium coordination compounds.

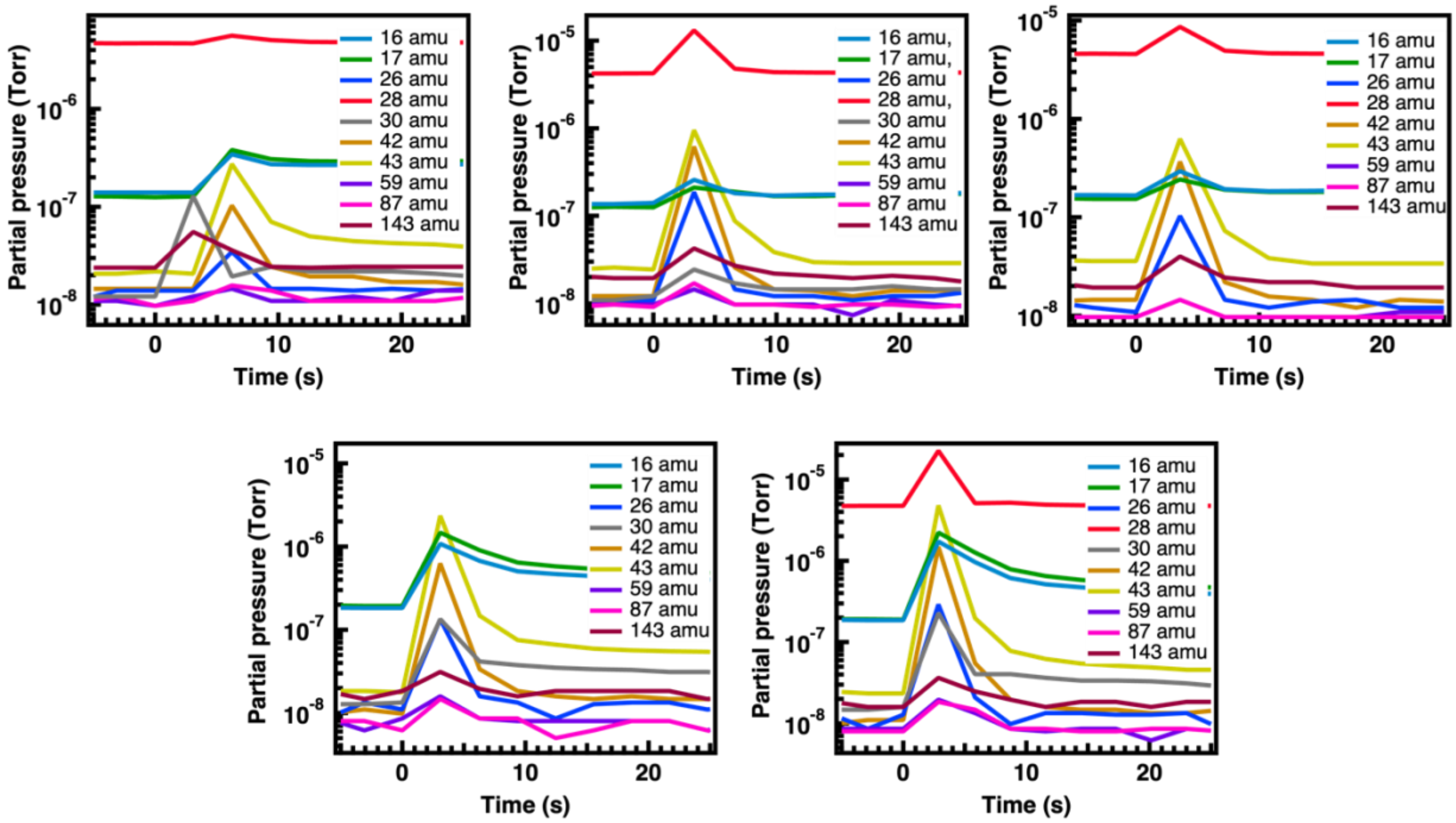

Figure S2. Partial pressure as a function of time for gaseous species following the irradiation of $UN_3(BIMA)_3$. Time = 0 corresponds to the time when the laser was turned on. The top row features, from left to right, the 10 W, then 20 W, then 20 W laser sequence on the same spot. The bottom row shows the 50 W scan on the left and the 100 W scan on the right.

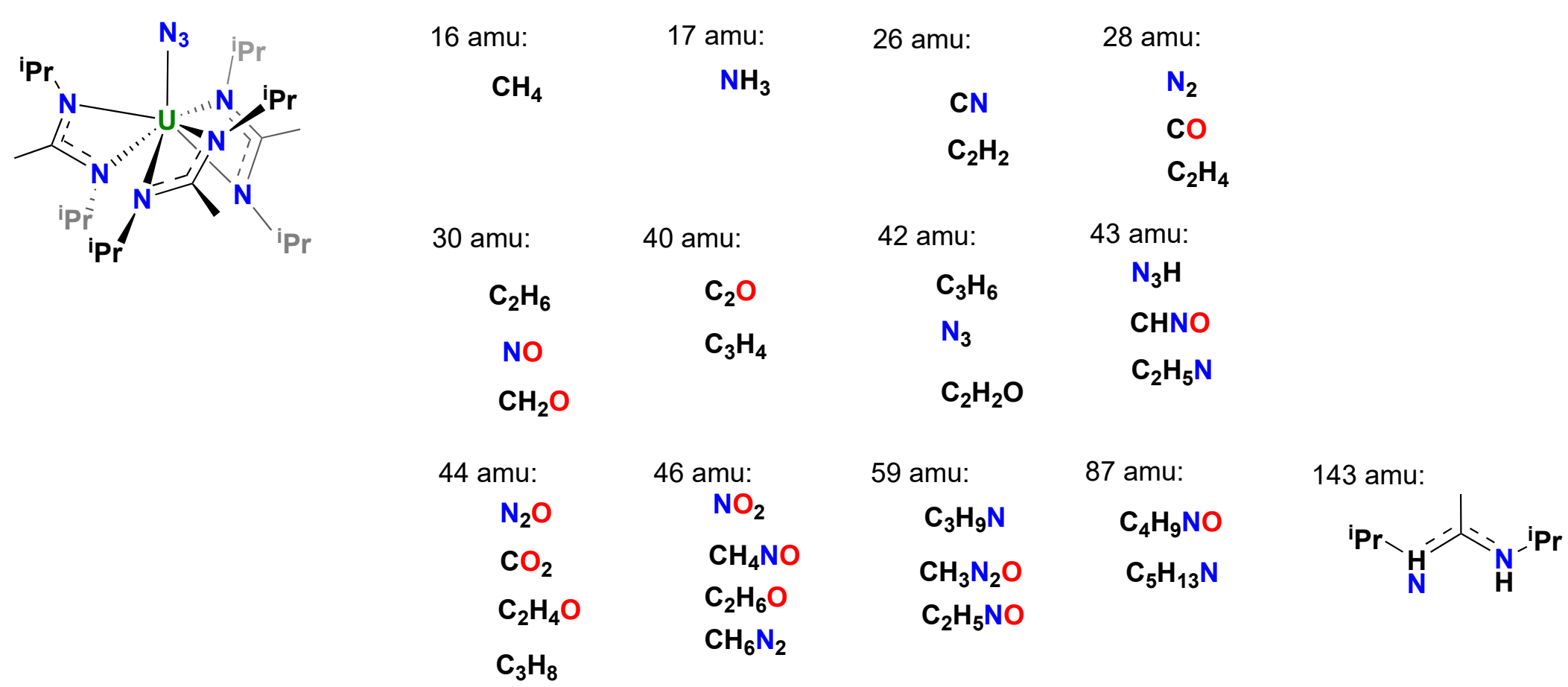

Figure S3. Predicted gaseous species for masses measured in $UN_3(BIMA)_3$ RGA experiments.

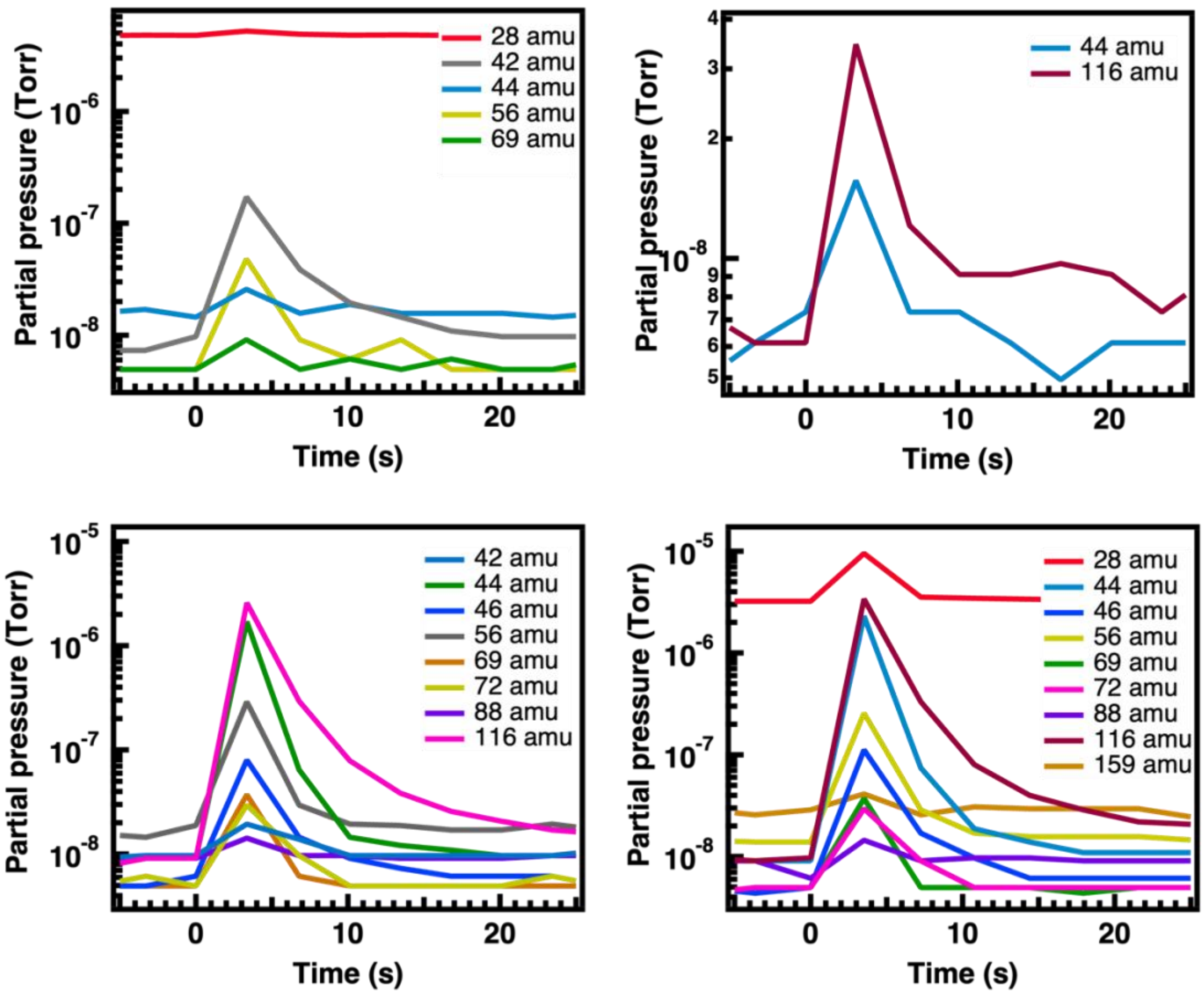

Figure S4. Partial pressure as a function of time for gaseous species following the irradiation of U(ITTA)$_4$. Time = 0 corresponds to the time when the laser was turned on. The top row features, the 10 W on the left and the first 20 W scan on the right; the second 20 W scan for this sample showed no changes in partial pressure, so has been omitted. The bottom row shows the 50 W scan on the left and the 100 W scan on the right.

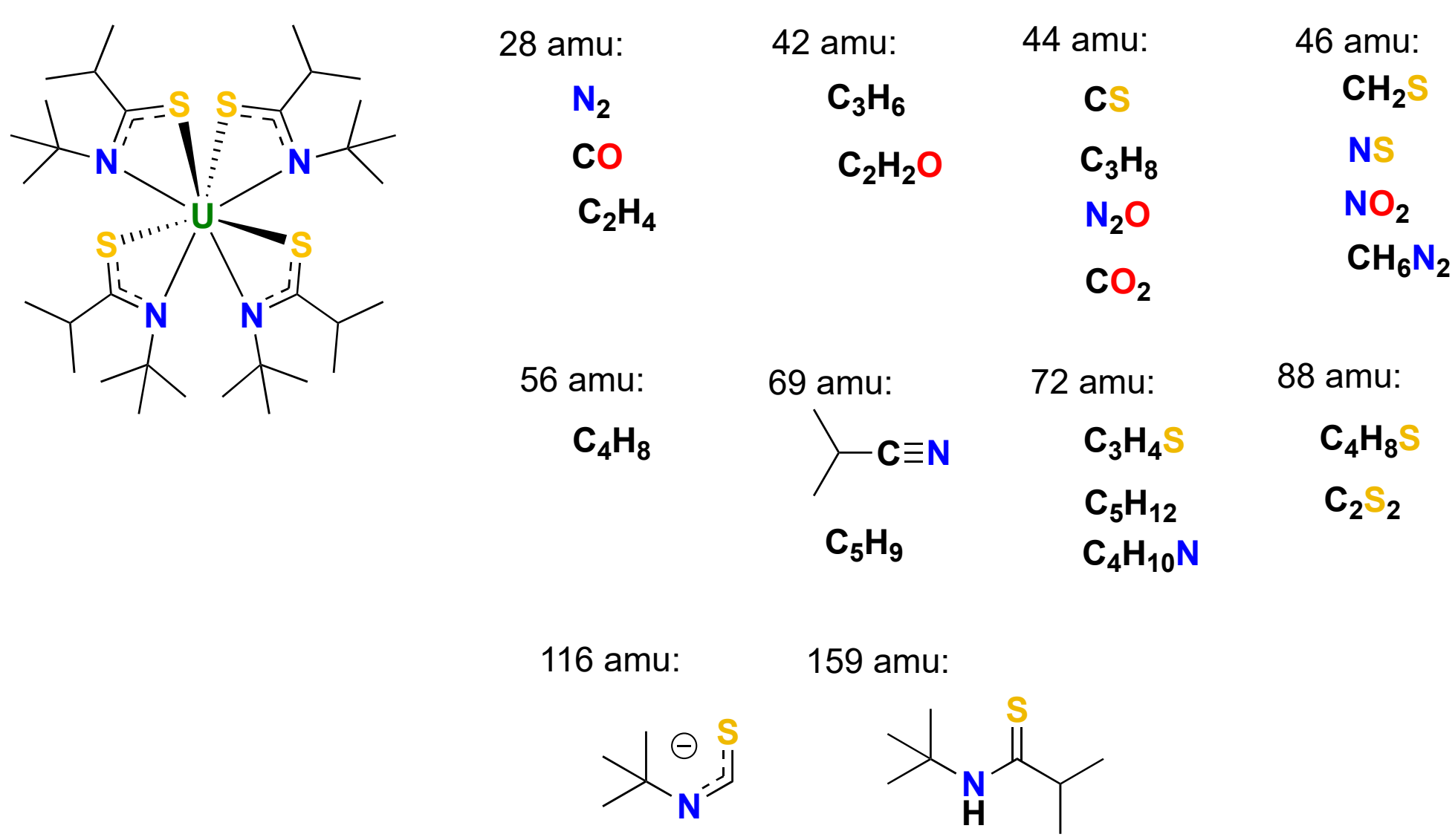

Figure S5. Predicted gaseous species for masses measured in U(ITTA)$_4$ RGA experiments.

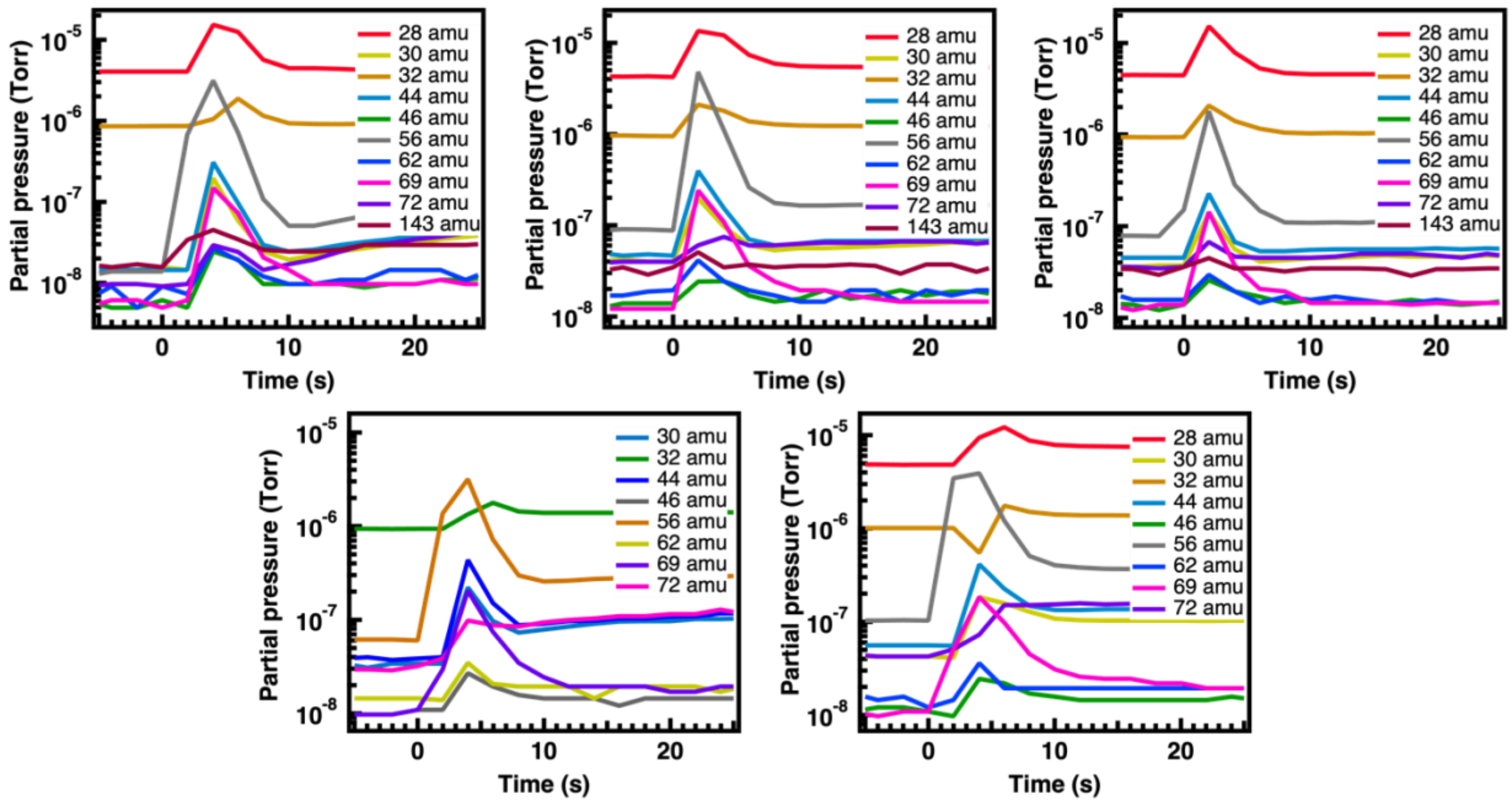

Figure S6. Partial pressure as a function of time for gaseous species following the irradiation of $U(ITA)_4$. Time = 0 corresponds to the time when the laser was turned on. The top row features, from left to right, the 10 W, then 20 W, then 20 W laser sequence on the same spot. The bottom row shows the 50 W scan on the left and the 100 W scan on the right.

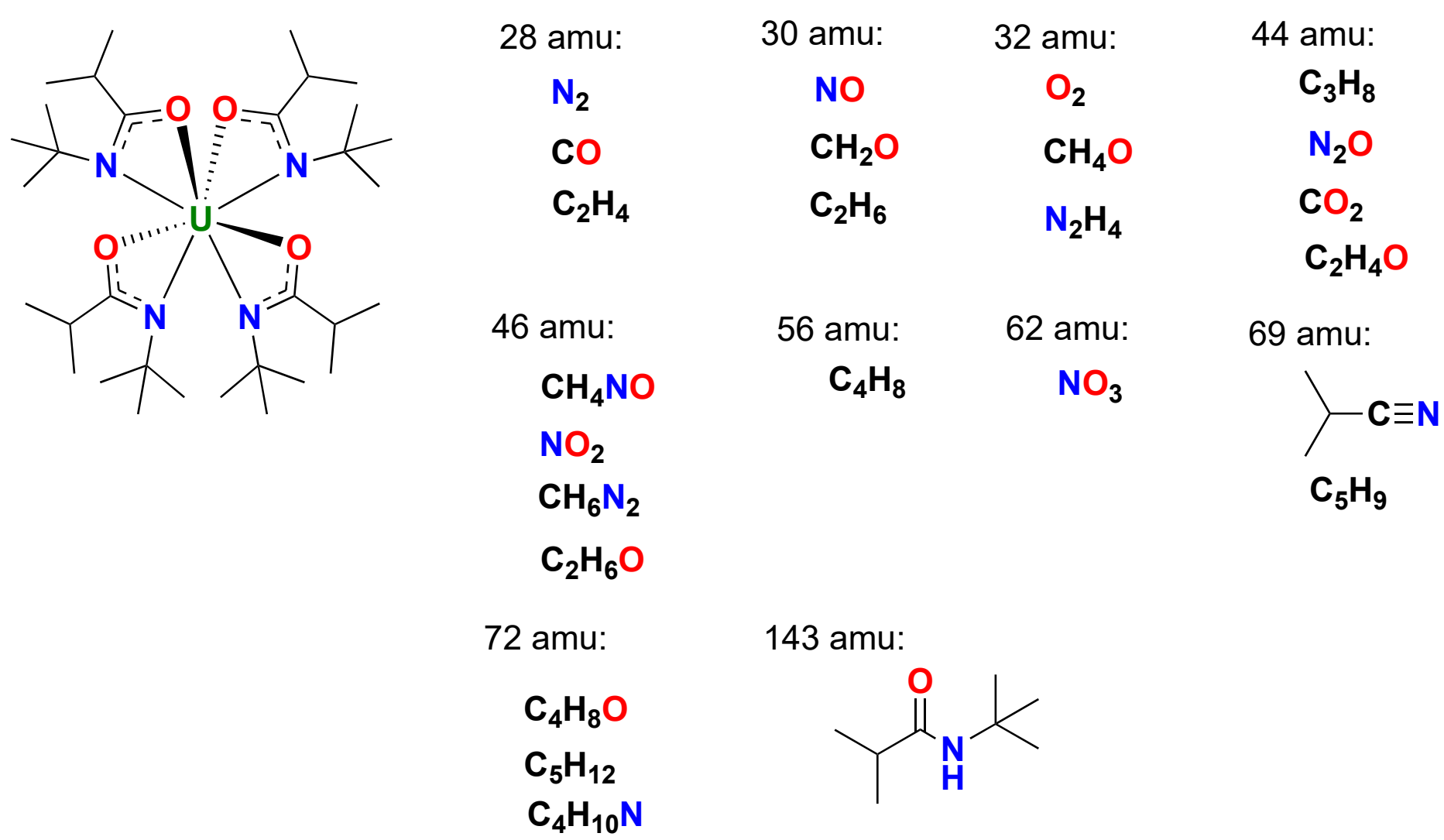

Figure S7. Predicted gaseous species for masses measured in $U(ITA)_4$ RGA experiments.

C. Powder X-Ray Diffraction

Table S1. Rietveld refinement for $UN_3(BIMA)_3$ after a 10 W, 10 W, 20 W, 50 W, 50 W pulse sequence. $\mathbf{X}^2$ for this fit is 0.995. Additional species, including UC and other carbonitride species do not improve the fit, or were calculated to 0% contribution upon addition to fitting.

Compound	Wt.%
UN	43
UN_2	35
UO_2	22

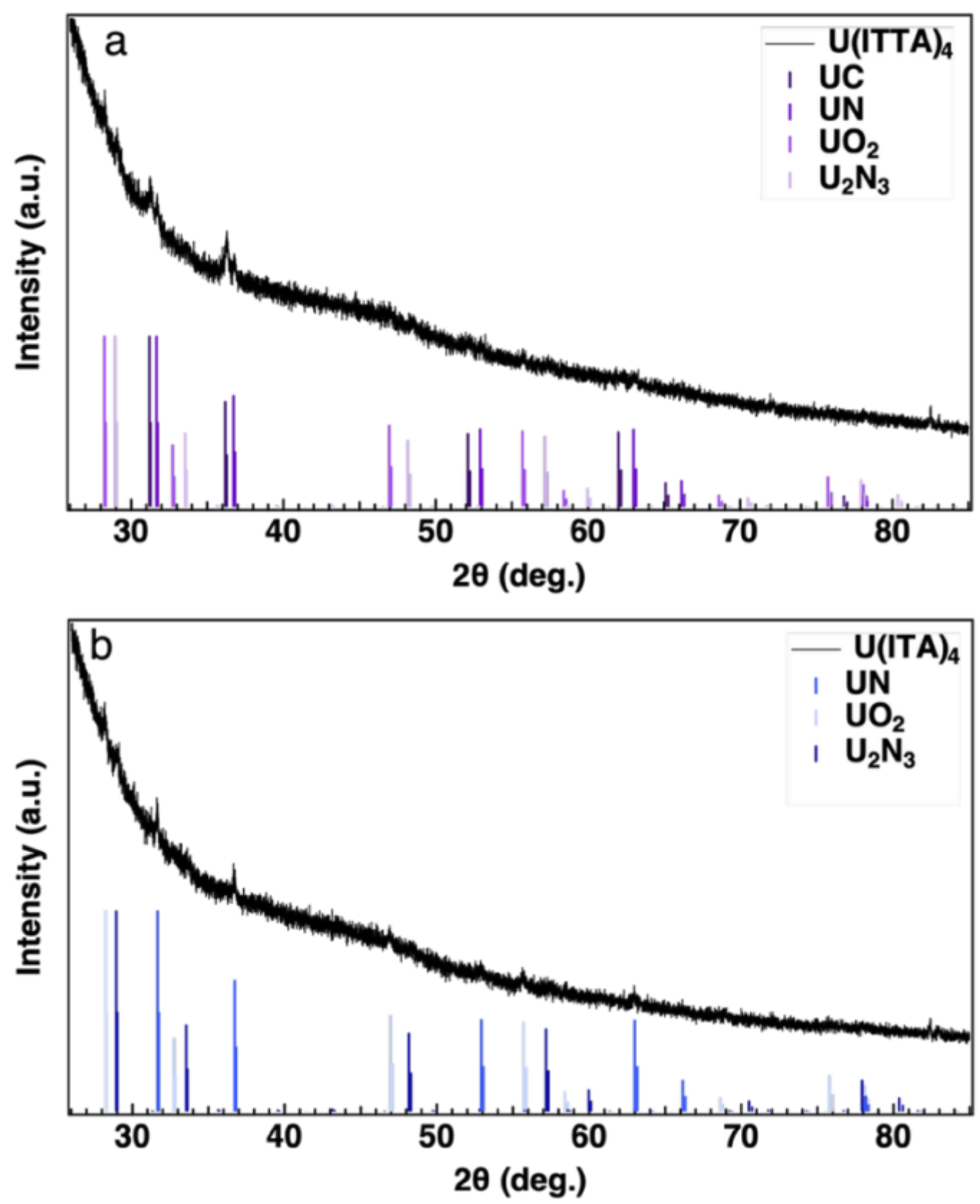

Figure S8. Powder X-ray diffraction for a) $U(ITTA)_4$ and b) $U(ITA)_4$ irradiated with two 10 W pulses, a 20 W pulse, and two 50 W pulses in sequence. Both powder patterns are presented in black, and are compared to their major products in purple ($U(ITTA)_4$) and blue ($U(ITA)_4$).

Table S2. Rietveld refinement for $U(ITTA)_4$ and $U(ITA)_4$ after a 10 W, 10 W, 20 W, 50 W, 50 W pulse sequence. $\mathbf{X}^2$ for the $U(ITTA)_4$ refinement is 1.164, and for $U(ITA)_4$ 0.989.

Sample	UC Wt.%	UN Wt.%	U_2N_3 Wt.%	UO_2 Wt.%
$U(ITTA)_4$	34	26	18	22
$U(ITA)_4$	N/A	45	16	39

D. References

(1) Straub, M. D.; Leduc, J.; Frank, M.; Raauf, A.; Lohrey, T. D.; Minasian, S. G.; Mathur, S.; Arnold, J. Chemical Vapor Deposition of Phase-Pure Uranium Dioxide Thin Films from Uranium(IV) Amidate Precursors. *Angew. Chem. Int. Ed.* **2019**, *58* (17), 5749–5753. https://doi.org/10.1002/anie.201901924.

(2) Kelly, S. N.; Russo, D. R.; Ouellette, E. T.; Roy, D.; Swift, A. J.; Boreen, M. A.; Smith, P. W.; Moreau, L. M.; Arnold, J.; Minasian, S. G. Formation of Uranium Disulfide from a Uranium Thioamidate Single-Source Precursor. *Chem. Sci.* **2024**, 10.1039.D4SC03422H. https://doi.org/10.1039/D4SC03422H.

(3) Straub, M. D.; Moreau, L. M.; Qiao, Y.; Ouellette, E. T.; Boreen, M. A.; Lohrey, T. D.; Settineri, N. S.; Hohloch, S.; Booth, C. H.; Minasian, S. G.; Arnold, J. Amidinate Supporting Ligands Influence Molecularity in Formation of Uranium Nitrides. *Inorg. Chem.* **2021**, *60* (9), 6672–6679. https://doi.org/10.1021/acs.inorgchem.1c00471.

(4) Ferrier, M. G.; Childs, B. C.; Silva, C. M.; Greenough, M. M.; Moore, E. E.; Swift, A. J.; Di Pietro, S. A.; Martin, A. A.; Jeffries, J. R.; Holliday, K. S. Unconventional Pathways to Carbide Phase Synthesis via Thermal Decomposition of UI_4(1,4-Dioxane)$_2$. *Inorg. Chem.* **2022**, *61* (44), 17579–17589. https://doi.org/10.1021/acs.inorgchem.2c02590.

(5) Ferrier, M. G.; Childs, B. C.; Silva, C. M.; Greenough, M. M.; Moore, E. E.; Erickson, K. A.; Monreal, M. J.; Colla, C. A.; Marple, M. A. T.; Winston, L. D.; Burks, J. N.; Martin, A. A.; Jeffries, J. R.; Holliday, K. S. Laser-Induced Thermal Decomposition of Uranium Coordination Compounds with Non-Oxidic Ligands to Produce Nitride and Carbide Materials. *Inorg. Chem.* **2024**, *63* (4), 1938–1946. https://doi.org/10.1021/acs.inorgchem.3c03591.

(6) Toby, B. H.; Von Dreele, R. B. GSAS-II: The Genesis of a Modern Open-Source All Purpose Crystallography Software Package. *J. Appl. Crystallogr.* **2013**, *46* (2), 544–549. https://doi.org/10.1107/S0021889813003531.
